# Supplementary material for: Characterization of Robertsonian and Reciprocal Translocations in Cattle through NGS
Source: Animals (Basel). 2023 Sep 26;13(19):3018. doi: 10.3390/ani13193018 (PMC10571785; doi:10.3390/ani13193018)

*Iannuzzi A et al. “Characterization of Robertsonian and reciprocal translocations in cattle by NGS sequencing”*

# Supplementary Information

1. **Calculation of the correction factor for the reads obtained on BTAX**

2. **Procedure used for the calculation of the shorter identifiable genomic fragment eventually involved in a rcp (simulation)**

**3. Identification of rcp translocation breakpoint**

**Calculation of the correction factor for the reads obtained on BTAX**

We computed the Le value using two WGS studies with material derived from blood extraction rather than microdissection. In this instance, we anticipate that the acquired reads will have a homogeneous distribution across all chromosomes. The results, however, revealed a higher concentration on the X chromosome, which generally indicates a number of reads greater than around 1.5 times.

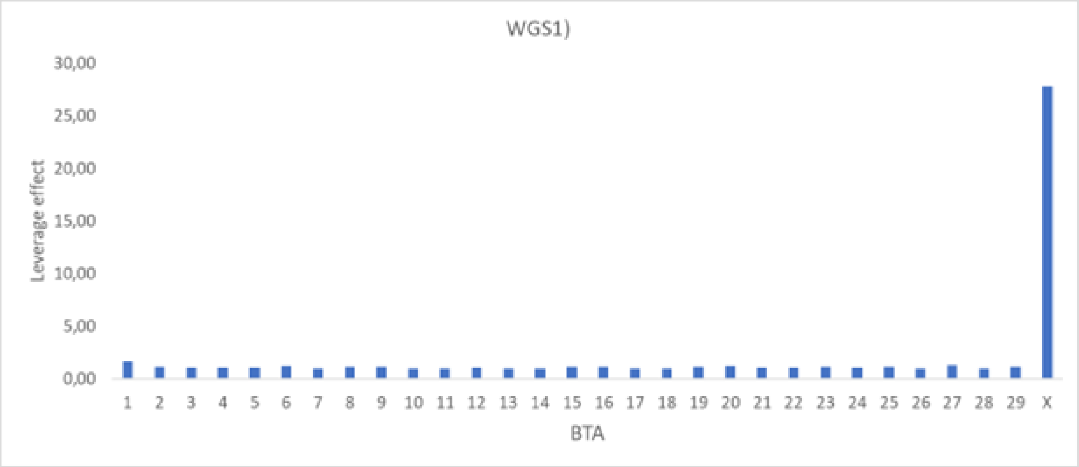


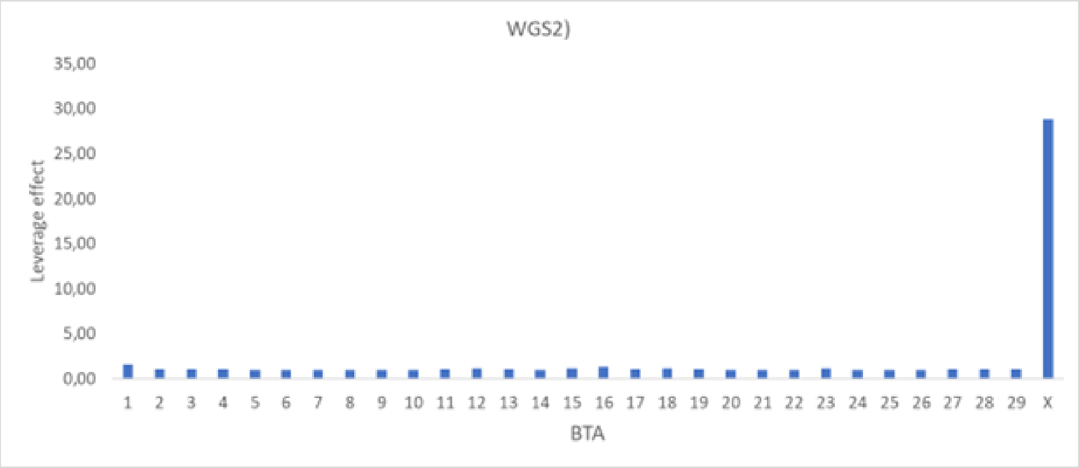


For this reason, in the Le calculation regarding rob26;29, rcp4;7 and rcp9;11 a factor *0.66 was applied to the reads obtained for BTAX.

**Procedure used for the calculation of the shorter identifiable genomic fragment eventually involved in a rcp (simulation)**

The data used for the construction of the regression shown in the article are the following:

| Anomaly[1] | BTA[2] | Reads Gen. (avg)[3] | Reads Inv (avg)[4] | Ratio[5] | Threshold reads[6] | Threshold Mb[7] |
|---|---|---|---|---|---|---|
| rob(26;29) | 26 | 3.024 | 4.949 | 1,6 | 217.000 | 43,8 |
| | 29 | 3.024 | 5.238 | 1,7 | 205.000 | 39,1 |
| rcp (4;7) | 4 | 2.678 | 104.263 | 38,9 | 2.100.000 | 20,1 |
| | 7 | 2.678 | 165.828 | 61,9 | 3.200.000 | 19,3 |
| rcp(9;11) | 9 | 2.008 | 17.811 | 8,9 | 750.000 | 42,1 |
| | 11 | 2.008 | 13.106 | 6,5 | 595.000 | 45,4 |

[1]: Anomaly examined
[2]: Chromosomes involved
[3]: Average of the reads/Mb value on the chromosomes not involved in the anomaly
[4]: Average of the reads/Mb value on the chromosomes involved in the anomaly
[5]: Ratio between 3 and 4 (Reads Inv / Reads gen).
[6]: Minimum number of total reads to reach a value of Le=4 (threshold of significance of a commonly accepted anomalous value).
[7]: Ratio between 6 and 4 (Threshold reads / Reads inv.). It represents the minimum identifiable size of a genomic fragment possibly involved in a RCP.

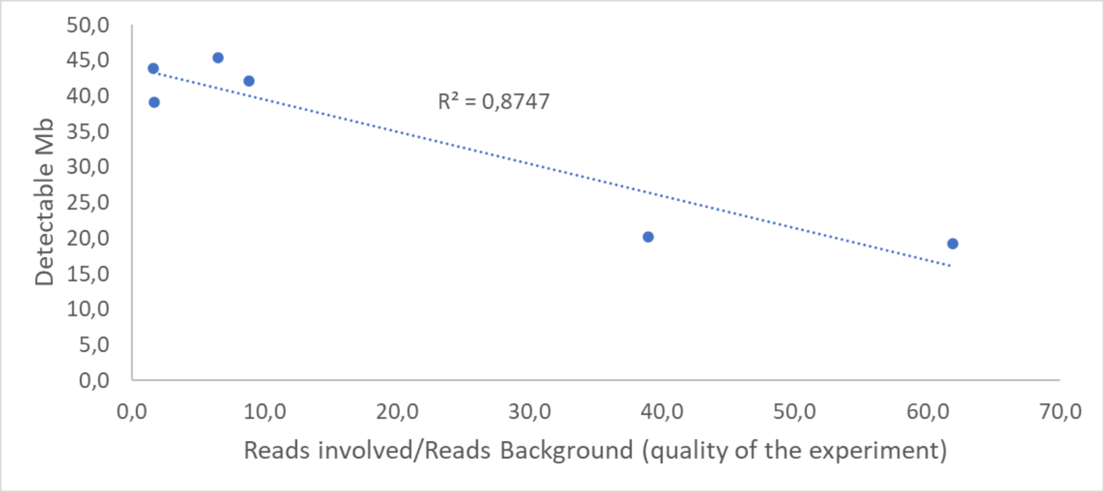


There is an important relationship between the quality of the experiment and the ability to identify small fragments.

## Identification of rcp translocation breakpoint

We analysed the distribution of reads on the sequenced derivative chromosomes to understand whether it was possible to obtain information regarding the breakpoint involved in the formation of rcps.
The first analysis was done on rcp4;7 (DeLorenzi et al., 2010).
The structure of this rcp is as follows:

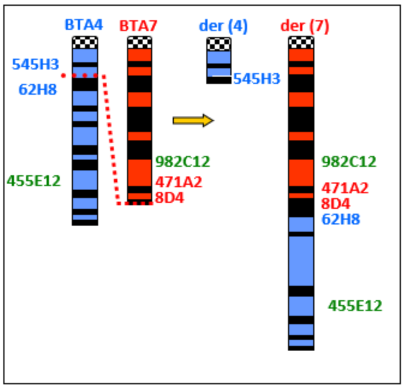


and therefore, considering the BTA4, the breaking point must be between its BAC 545H3 and 62H8, or between approximately 15.7 and 34.9 Mb of the chromosome length. Initially we analyzed the distribution of the reads on BTA4 (remember that der7 was sequenced which does not contain the centromeric part of BTA4) and the result obtained was the following:

| BTA4 bp | Reads |
|---|---|
| 1-10,000,000 | 13.779 |
| 10,000,000-20,000,000 | 9.116 |
| 20,000,000-30,000,000 | 12.448 |
| 30,000,000-40,000,000 | 1.096.055 |
| 40,000,000-50,000,000 | 1.466.394 |
| 50,000,000-60,000,000 | 1.408.162 |
| 60,000,000-70,000,000 | 1.480.477 |
| 70,000,000-80,000,000 | 1.516.629 |
| 80,000,000-90,000,000 | 1.476.238 |
| 90,000,000-100,000,000 | 1.364.242 |
| 100,000,000-110,000,000 | 1.378.057 |
| 110,000,000-120,829,699 | 1.376.476 |

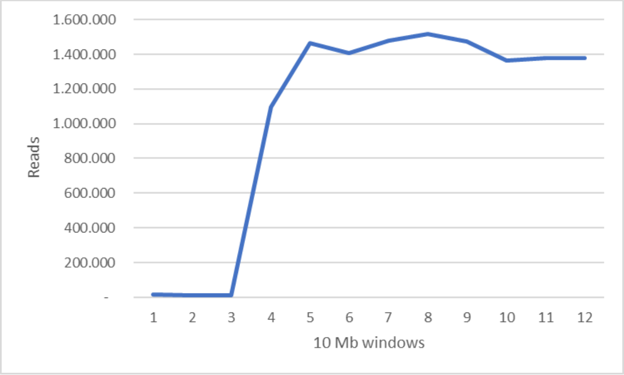


it is clear that the cenomeric region has significantly fewer reads as expected. we therefore deepened the analysis of this region by decreasing the analysis window and the final result is the following:

| BTA4 bp | | |
|---|---|---|
| from | to | Reads |
| 33.310.000,00 | 33.320.000,00 | 0 |
| 33.320.000,00 | 33.330.000,00 | 10 |
| 33.330.000,00 | 33.340.000,00 | 0 |
| 33.340.000,00 | 33.350.000,00 | 1 |
| 33.350.000,00 | 33.360.000,00 | 0 |
| 33.360.000,00 | 33.370.000,00 | 0 |
| 33.370.000,00 | 33.380.000,00 | 3 |
| 33.380.000,00 | 33.390.000,00 | 0 |
| 33.390.000,00 | 33.400.000,00 | 3 |
| 33.400.000,00 | 33.410.000,00 | 0 |
| 33.410.000,00 | 33.420.000,00 | 0 |
| 33.420.000,00 | 33.430.000,00 | 2 |
| 33.430.000,00 | 33.440.000,00 | 36 |
| 33.440.000,00 | 33.450.000,00 | 1786 |
| 33.450.000,00 | 33.460.000,00 | 2568 |
| 33.460.000,00 | 33.470.000,00 | 1468 |
| 33.470.000,00 | 33.480.000,00 | 631 |
| 33.480.000,00 | 33.490.000,00 | 3107 |
| 33.490.000,00 | 33.500.000,00 | 924 |

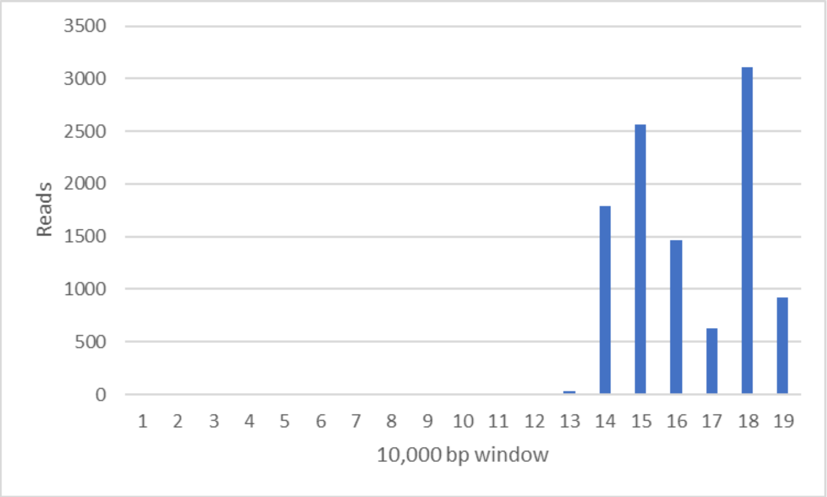

It therefore appears clear that the breaking point on the BTA4 must be between 33,430,000 bp and 33,450,000, a result that perfectly coincides with what was obtained from the FISH analyzes present in the original publication.

As regards the breaking point on BTA7, this must be present in an extremely terminal position, and in any case beyond the base 110,278,961, i.e. the terminal position of the BAC 8D4. The result obtained suggests that this B.P. must be present very close to the telomere, as a decrease in reads in the terminal megabases of the chromosome could not be appreciated.

| BTA7 bp | | |
|---|---|---|
| **from** | **to** | **Reads** |
| 103.000.000 | 104.000.000 | 142.171 |
| 104.000.000 | 105.000.000 | 137.265 |
| 105.000.000 | 106.000.000 | 162.401 |
| 106.000.000 | 107.000.000 | 175.609 |
| 107.000.000 | 108.000.000 | 136.453 |
| 108.000.000 | 109.000.000 | 169.222 |
| 109.000.000 | 110.000.000 | 147.442 |
| 110.000.000 | 111.000.000 | 159.441 |
| 111.000.000 | 112.000.000 | 126.446 |
| 112.000.000 | 112.638.659 | 115.261 |

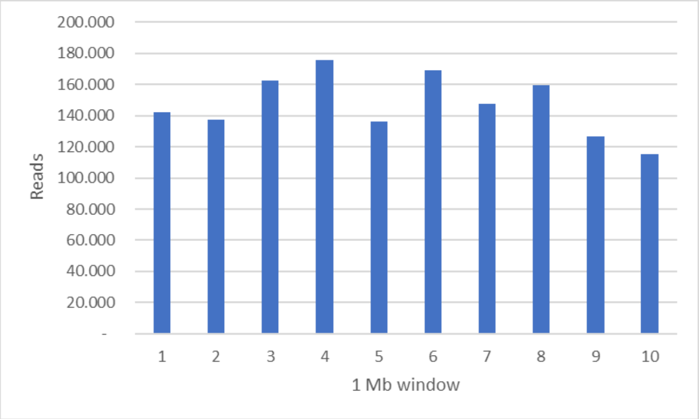

Supplement: Supplementary file 1 [file animals-13-03018-s001.zip › animals-2550758-supplementary.pdf]
